# Single Image Super-Resolution via Wide-Activation Feature Distillation Network

**DOI:** 10.3390/s24144597

**Published:** 2024-07-16

**Authors:** Zhen Su, Yuze Wang, Xiang Ma, Mang Sun, Deqiang Cheng, Chao Li, He Jiang

**Affiliations:** 1 School of Information and Control Engineering, China University of Mining and Technology, Xuzhou 221116, China; suzhen83@163.com (Z.S.); wangyuze@cumt.edu.cn (Y.W.); maxiang00@cumt.edu.cn (X.M.); sunmang@cumt.edu.cn (M.S.); chengdq@cumt.edu.cn (D.C.); lichao15755876764@163.com (C.L.); 2 Science and Technology Bureau of Jining City, Shandong Province, Jining 272000, China; 3 State Grid Nanjing Automation Co., Ltd., Nanjing 210032, China

**Keywords:** super-resolution, wide activation, feature distillation, dual-path learning, lightweight

## Abstract

Feature extraction plays a pivotal role in the context of single image super-resolution. Nonetheless, relying on a single feature extraction method often undermines the full potential of feature representation, hampering the model’s overall performance. To tackle this issue, this study introduces the wide-activation feature distillation network (WFDN), which realizes single image super-resolution through dual-path learning. Initially, a dual-path parallel network structure is employed, utilizing a residual network as the backbone and incorporating global residual connections to enhance feature exploitation and expedite network convergence. Subsequently, a feature distillation block is adopted, characterized by fast training speed and a low parameter count. Simultaneously, a wide-activation mechanism is integrated to further enhance the representational capacity of high-frequency features. Lastly, a gated fusion mechanism is introduced to weight the fusion of feature information extracted from the dual branches. This mechanism enhances reconstruction performance while mitigating information redundancy. Extensive experiments demonstrate that the proposed algorithm achieves stable and superior results compared to the state-of-the-art methods, as evidenced by quantitative evaluation metrics tests conducted on four benchmark datasets. Furthermore, our WFDN excels in reconstructing images with richer detailed textures, more realistic lines, and clearer structures, affirming its exceptional superiority and robustness.

## 1. Introduction

Single image super-resolution (SISR), as an important research field in computer vision, is dedicated to accurately reconstructing a high resolution (HR) image from one or more low resolution (LR) images. SISR models show a wide range of application potentials in the fields of image retrieval [1,2], computational photography [3,4,5,6,7], and intelligent mining [8]. Currently, mainstream SISR algorithms can be summarized into three main categories: interpolation-based algorithms [9], which estimate high-resolution images by calculating the relationship between the pixels; reconstruction-based algorithms [10], which focus on recovering high-resolution details by using a priori information or the constraints of the image itself; and statistical learning-based algorithms [11], which learn the mapping relationship from LR to HR with the help of a large amount of training data, in order to achieve more accurate reconstruction of high-resolution images.

With the rapid development of deep learning technology and the significant increase in computer computing power, SISR algorithms based on deep neural networks have become increasingly popular. The SRCNN [12], with its revolutionary contribution, was the first to incorporate convolutional neural networks into the SISR model, which skillfully decomposes the super-resolution task into three core steps: shallow feature extraction, nonlinear feature mapping, and fine image reconstruction. This approach has been widely acclaimed for its excellent performance and concise architectural design. However, the need for the SRCNN to uplift the image prior to feature extraction undoubtedly adds unnecessary computational burden and affects the overall efficiency. The VDSR [13] algorithm, on the other hand, employs the idea of residual learning and focuses on learning the residual layers in the image, which dramatically improves the performance of the model. This approach not only improves the quality of the reconstructed image, but also optimizes the computational efficiency. However, its potential challenge is that the accurate learning of residual layers may require more complex network structures and training strategies. The LapSRN [14] algorithm borrows the structure of the Laplace pyramid and extends the asymptotic design concept of VDSR. This innovation not only improves the accuracy of image reconstruction, but also enhances the adaptability of the algorithm to images of different scales. However, the complex network structure and multi-scale processing strategy also bring challenges in training and consumption of computational resources. The ESPCN [15] algorithm is another milestone in the field of SISR. It introduces pixel shuffling in the sub-pixel domain, a unique design that eliminates a large number of redundant steps in the pre-feature extraction stage, making the algorithm more efficient and straightforward. This innovative technique has become a standard element in modern SISR models, revolutionizing the SISR task. However, pixel shuffling techniques may also introduce additional noise or distortion in some specific scenarios. The EDSR [16] algorithm explores the SISR model in more depth. It decisively removes the batch normalization layer and experimentally verifies the effectiveness of this decision in the SISR task. This simplification not only improves the performance of the model but also reduces the computational complexity. However, it also requires the algorithm to adjust the learning rate and optimization strategy more finely during the training process to ensure the stability and generalization ability of the model.

With the increasing pursuit of image reconstruction performance, the number of parameters of network models has also exploded, which undoubtedly poses great challenges to the training and inference process of the models, which, in turn, limits the wide deployment of these models in practical applications. To overcome these limitations, researchers have focused on the development of lightweight SISR networks, with a view to improving training efficiency and realizing efficient inference. Hui et al. have made remarkable progress in this area by introducing the information distillation technique and proposing the IDN [17] model. The model cleverly utilizes hierarchical features to effectively improve the performance of the model by processing the current feature map separately. However, the IDN [17] model is slightly stretched when dealing with multi-scale information and has high computational complexity. To compensate for this deficiency, Hui et al. subsequently introduced the IMDN [18] model, which is capable of capturing more multi-scale detail information. Unfortunately, multi-scale information fusion also leads to the problem of too many network parameters and high computational complexity [19,20].

The above methods have successfully achieved a good balance between reconstruction accuracy and model complexity. However, these methods tend to ignore the effect of network width on reconstruction performance, and many of them still suffer from limited feature extraction and under-utilization of features.

To solve the above problems, Liu et al. creatively put forward the RFDN [21], which is characterized by superior performance, high efficiency, and a small number of parameters. This network significantly reduces computational complexity and the number of parameters by introducing residual connection and feature distillation mechanisms. Specifically, the RFDN uses pointwise convolution (i.e., 1 × 1 kernel size convolution) instead of channel segmentation, effectively reducing the number of channels and parameters. In addition, the model introduces a shallow residual learning module to replace the coarse-grained residual connectivity in the IMDN, which realizes the improvement of network performance without increasing the number of parameters. Although the feature distillation technique reduces the number of parameters and improves the network performance, it requires a correspondingly higher hardware environment, which limits the inference speed of RFDNs to some extent. Also, the large number of redundant convolutional operations in RFDNs increases the computational burden of the model, making deployment in resource-constrained environments more challenging.

In this paper, a novel wide-activation feature distillation network (WFDN) is introduced, based on the wide-activation network [22] and the feature distillation network [18,21]. The main contributions of this study can be summarized as follows:

- Dual-path parallel network structure: single-path networks usually rely on a single feature extraction method, which makes it difficult to fully extract and utilize the deep features in the image. To overcome the limitations of single-path networks, a dual-path parallel network structure inspired by the DPN [23] is used in this study. In addition, global hopping connections are used to accelerate the information flow, improve the performance, and speed up the convergence of the network.
- Wide-activation residual feature distillation module: in order to comprehensively acquire high-frequency feature information, the concept of wide activation is introduced in the residual feature distillation module, and a lightweight attention mechanism is used to learn high-frequency spatial location information. This integration enhances the feature representation capability of the network without additional parameters or computational overheads.
- Gated feature fusion module: in order to alleviate the problem of the redundancy of key information caused by undifferentiated element-summing operations, a gated feature fusion module is introduced in the feature fusion stage. This module generates different gating weights to process the feature information from the upper and lower branches, which enhances the complementarity between the feature information and ultimately improves the reconstruction effect of the fused features.

In this paper, a novel wide-activation feature distillation network (WFDN) is proposed for enhancing the performance of SISR. The introduction section presents the background, technological advances, and challenges in the field of SISR and outlines the main innovations of the WFDN, including the dual-path parallel structure, the wide-activation residual feature distillation module, and the gated feature fusion module. The related work section reviews the existing research on lightweight SISR models, feature distillation networks, attention mechanisms, and super-resolution image quality assessment techniques. The proposed method section describes in detail each key module of the WFDN and its design ideas. The experimental results and analysis section verifies the superiority of the WFDN on multiple datasets, through metrics such as the PSNR and the SSIM, and evaluates its visual effect through subjective scoring methods. The ablation experiment section further validates the effectiveness of each module. The conclusion section summarizes the significant advantages of the WFDN in improving image reconstruction performance, reducing the number of model parameters and computational complexity, and demonstrates its potential and application prospects in SISR tasks.

## 2. Related Work

### 2.1. Lightweight SISR Models

Lightweight SISR models bring the possibility of high-quality image enhancement to mobile devices and fulfill users’ needs for image quality improvement. With the advancement of computing hardware performance and the development of deep learning algorithms, it has become possible to design lightweight models and maintain high-quality super-resolution results. First, the SRCNN [12], as a preliminary exploration of deep convolutional neural networks in the field of super-resolution, has the advantage of being able to learn and directly map end-to-end functional relationships, which, in turn, enables rapid and high-quality reconstruction of images. However, due to the relative simplicity of the network structure, it may be slightly stretched when dealing with complex textures and fine details. Immediately after that, EDSR [16] significantly improves the detail rendering and clarity of images by skillfully improving the baseline architecture of the deep super-resolution network. Its uniqueness lies in the fact that it greatly improves the efficiency of information transfer through residual connections and dense connections. However, this design brings a corresponding increase in computational complexity when dealing with large size images. The DRCN [24], on the other hand, skillfully realizes deep super-resolution through recursive convolutional layers and successfully captures more detailed information in images. However, its recursive architecture improves performance while leading to a slowdown in training speed and an increase in memory consumption, which becomes a limitation in some application scenarios. The DRRN [25] further fuses residual concatenation and recursive architecture to achieve a significant improvement in super-resolution performance. It performs particularly well in recovering image details, but faces the same challenge of excessive memory consumption when dealing with large-size images. The CARN [26] skillfully balances the super-resolution effect with computational efficiency by cascading residual networks. However, it occasionally introduces slight artifacts when processing complex textures, which, to some extent, impacts the overall quality of the image. Finally, SMSR [27] achieves efficient inference in resource-constrained environments by introducing sparsity. However, this strategy may also introduce a partial loss of detail information when dealing with highly compressed images.

### 2.2. Distillation Network-Based SISR Models

In the field of lightweight image super-resolution reconstruction, the information distillation technique is undoubtedly a key force in improving model performance and reducing computational cost. Hui et al. pioneered the IDN [17], which skillfully utilizes the information distillation mechanism to realize lightweight SISR reconstruction. The advantage of the IDN [17] lies in the fact that it significantly improves training speed through the information distillation technique, so that the model can converge quickly, and it greatly accelerates the process of model training. However, as the technology has progressed, the IDN [17] has also shown its limitations: due to its relatively high computational complexity, it encounters a bottleneck when processing multi-scale information, which may make its performance unsatisfactory on certain detail-rich images, especially when processing high-resolution images, and its performance may be significantly affected. In order to overcome the limitations of IDNs in processing multi-scale information, Hui et al. subsequently proposed the IMDN [18]. The IMDN [18] takes a step further from the IDNs in that it is able to utilize the information at different scales more efficiently, which significantly improves the effectiveness of super-resolution reconstruction. This innovation enables the IMDN [18] to more accurately restore the details of an image while maintaining its lightweight nature, and it especially excels in handling multi-scale information. However, even though the IMDN has improved in performance, it still faces certain computational complexity challenges. Subsequently, Liu et al. proposed the RFDN [21], a lightweight image super-resolution network. The RFDN [21] is unique in that it significantly reduces computational complexity and the number of parameters by incorporating residual concatenation and feature distillation techniques to achieve efficient super-resolution reconstruction. This design allows the RFDN [21] to achieve excellent reconstruction results at low computational cost while remaining lightweight. Although the RFDN [21] has achieved remarkable results in reducing computational costs, its performance may be somewhat limited when dealing with high-resolution images, especially those containing complex textures and details. To further enhance the model performance, Kong et al. introduced the RLFN [28], a network structure optimization method based on a lightweight feature fusion module. The highlight of the RLFN [28] is that it significantly improves the representation capability of the model through feature fusion techniques, which enables better restoration of image details. This design allows the RLFN [28] to excel in handling image details, especially when dealing with low-resolution images. However, when confronted with high-resolution images, the RLFN [28] still has some limitations and needs further optimization and improvement to better cope with more complex textures and details in high-resolution images. To address these challenges, Li et al. drew inspiration from the RFDN and proposed the BSRN [29]. The BSRN [29] improves model representation by introducing blueprint-separable convolution, which enables the model to better cope with the super-resolution reconstruction task of high-resolution images while maintaining the lightweight property. This improvement makes the BSRN [29] perform better in processing high-resolution images, especially when dealing with complex textures and details. However, the BSRN still faces certain computational complexity challenges when dealing with very high-resolution images. Recently, Huang et al. proposed the DLSR [30] model, a new approach to exploring lightweight SISR models based on the differentiable neural architecture search method. The DLSR [30] model improves performance by virtue of its information refinement technique and minimizes computational complexity by optimizing the network structure. This enables the DLSR [30] model to have low computational cost while maintaining high performance. However, the differentiable neural architecture search method requires longer training time and computational resources, which is a potential shortcoming of the DLSR [30] model. In addition, DLSR [30] models require additional tuning and optimization when dealing with specific types of images or tasks.

### 2.3. Attention Mechanisms in the SISR Models

The attention mechanism in deep learning, an advanced tool for modeling human visual and cognitive systems, gives neural networks the unique ability to accurately and efficiently focus on key parts of the input data. The introduction of this mechanism allows neural networks to automatically learn and focus on the core information in the input, which significantly improves the performance and generalization of the model. Nonlocal attention [31] specializes in capturing dependencies in sequences that span long distances, providing strong support for processing complex data. Meanwhile, channel attention [32,33] optimizes inter-channel relationships in deep learning by learning the importance weight of each channel in a fine-grained manner, enabling the model to more accurately allocate attention to channels that are sensitive to key information, which greatly improves the model’s ability to model image channel relationships. Furthermore, spatial transformer attention [34] skillfully combines geometric transformations with the attention mechanism to provide a more fine-grained allocation of attention for spatial modeling and specific regions, enabling the model to more accurately capture key information in the image. Second-order attention [35], on the other hand, further enhances the model’s modeling ability and contextual awareness by considering the interactions between features in depth, enabling the model to understand the intrinsic structure of the data in a more comprehensive way. On this basis, enhanced spatial attention [36], as a more advanced mechanism, combines spatial and channel attention methods, and is able to simulate the relationship between space and channel at the same time, which injects new vitality into the improvement of model performance. In addition, pixel attention [37] is even more refined to the pixel level, allowing the model to accurately weight each pixel position in the image, thus focusing on the image details in greater detail and enhancing the ability to perceive subtle changes. Finally, the self-attention [38,39] mechanism effectively captures long-range dependencies in sequences by facilitating interactions between elements at different locations, making the model more comfortable in understanding global relationships in sequences.

However, while these attention mechanisms have made significant progress in areas such as image super-resolution reconstruction, they each have some limitations. For example, while the channel attention mechanism used in RCAN [33] improves the image reconstruction effect, it may neglect the local details in the image, resulting in the omission of some important features. The enhanced spatial attention mechanism in RFANet [36] captures spatial information, but it may be limited to a local-context range, making it difficult to deal with global relationships efficiently. The pixel attention mechanism in PAN [37] fine-tunes the importance of features, but it may increase computational cost, affecting the global relationships in PAN [37] and the network inference speed, especially when dealing with large-scale images. The self-attention mechanism in SwinIR [39] can deal with long-range dependencies, but may increase computational complexity when dealing with large-scale images, leading to a significant increase in training and inference costs.

### 2.4. Image Quality Evaluation Methods in SISR Models

Super-resolution image quality assessment techniques play a crucial role in the field of SISR, and they are used to measure and compare the performance of different SISR algorithms, helping researchers and developers to improve their models and select the optimal solution. In recent years, many new techniques have emerged, including LPIPS [40], MFFN [41], RISTRA [42], CN-BSRIQA [43], multi-branch networks for reference-free image quality assessment [44], and the graph-represented distribution similarity index for full-reference image quality assessment [45]. These techniques significantly improve the accuracy of image quality assessment and the reconstruction performance of models through deep learning of perceptual similarity metrics, advanced network architectures and fusion mechanisms, cascaded network structures, and graph structure analysis.

These new techniques demonstrate significant advantages in the field of image quality assessment. For example, LPIPS [40] captures high-level semantic information and visual features by comparing the depth features of an image, which is more reflective of visual perception similarity than the traditional PSNR and SSIM [46]. MFFN [41] utilizes a multilevel feature fusion network to improve the reconstruction quality and detail performance of the model by fusing features at different levels. RISTRA [42] combines a relative evaluation mechanism with a recursive image super-resolution transformer model, which improves the detail level of image reconstruction. CN-BSRIQA [43] is a reference-free super-resolution image quality assessment method, which realizes high-precision assessment through the structure of a cascade network, and which is suitable for practical application scenarios in which the reference image cannot be obtained. The multi-branch multilayer feature fusion network [44] improves the accuracy of reference-free image quality assessment by simultaneously processing image features at different levels. The graph-represented distribution similarity index [45], on the other hand, provides a more detailed full-reference image quality assessment, which is especially outstanding in assessing detail fidelity and global structural consistency.

Although these new techniques have made significant progress in improving the accuracy of image quality assessment, they have some drawbacks. First, these methods usually require high computational resources, leading to increased computational costs. Second, the complexity of the models is large, which increases the difficulty of implementation and deployment. In addition, the generalizability of these techniques in different application scenarios may be insufficient to fully replace the traditional methods. Therefore, for this paper we chose to use the PSNR and the SSIM [46] as quantitative metrics, which are traditional methods that are relatively simple but have better interpretability and computational efficiency, and have been widely accepted and used.

## 3. Proposed Method

**Figure 1:**
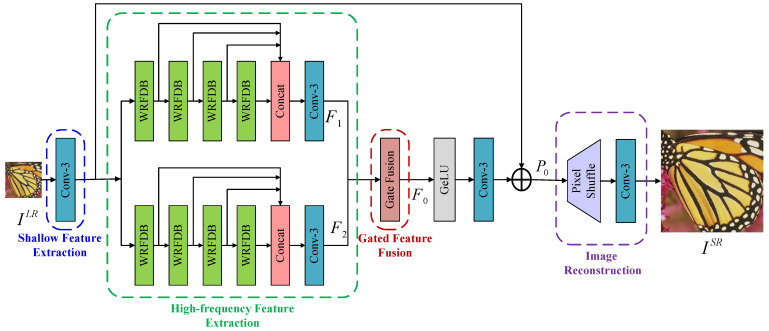
Schematic diagram of the WFDN.

In the network structure design phase, this study optimizes the conventional DPN [23] and proposes a novel wide-activation feature distillation network for SISR. This model is based on wide-activation and feature distillation networks. The overall architecture is illustrated in Figure 1. The network model comprises four sub-modules. Firstly, the shallow feature extraction module, depicted by a blue-dotted-line frame, employs a 3×3 convolution layer to extract shallow features. These features are then forwarded to the upper and lower branches for further extraction of high-frequency features. Secondly, the high-frequency feature extraction module, represented by the green-dotted-line frame, combines features across different levels, using both global and local connections. It effectively harnesses depth features from all levels while extracting high-frequency features from the image. This module, which integrates the wide-activated residual block and the attention mechanism, serves as a fundamental unit for basic feature extraction within the network structure. Furthermore, the gated feature fusion module, denoted by the red-dotted-line box, adaptively assigns different weights to the features extracted from the upper and lower branches. It subsequently fuses these features and introduces non-linearity through the ReLU activation function and a 3×3 convolutional layer. Lastly, the upsampling reconstruction module, depicted by the purple-dotted-line frame, primarily consists of a post-upsampling reconstruction process. This process involves a sub-pixel convolutional layer [15] followed by a 3×3 convolutional layer. The post-upsampling technique enables the network to learn adaptive upsampling, thereby facilitating the reconstruction of the final HR image.

### 3.1. Shallow Feature Extraction

Shallow feature extraction is a fundamental step in neural networks. During this stage, basic features of images are captured using a 3 × 3 convolutional layer, and they are referred to as shallow features. Subsequently, the extracted shallow features are propagated to the upper and lower branches of the network for the subsequent extraction of high-frequency features. Specifically, this process can be described using the following mathematical formula:

(1) $$F_{S}=H_{conv3}\left(I^{LR}\right)$$

where $I^{LR}$ represents the low-resolution image, $H_{conv3}\left(\right)$ denotes the convolution operation with a 3 × 3 kernel, and $F_{S}$ represents the extracted shallow features.

### 3.2. High-Frequency Feature Extraction

#### 3.2.1. Enhanced Spatial Attention and Contrast-Aware Channel Attention

In this network, the utilization of enhanced spatial attention (ESA) [36] primarily occurs at the termination of the residual block, with a specific emphasis on high-frequency spatial features. Given that low-frequency information, which exhibits relatively smooth spatial positioning in LR images, differs significantly from high-frequency detailed information encompassing edges and contours, the incorporation of this module directs heightened attention towards regions harboring high-frequency spatial information. Consequently, this approach facilitates the reconstruction of images with enhanced texture clarity. Notably, the module possesses a substantial receptive field while maintaining a lightweight parameter and computational load.

**Figure 2:**
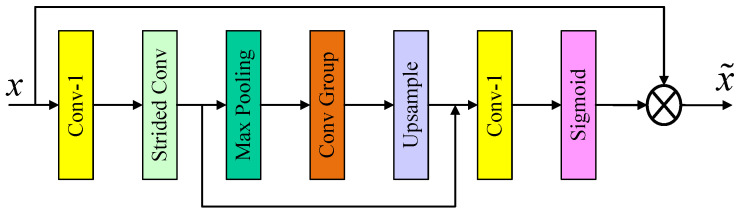
Schematic diagram of ESA.

Initially, the module employs a 1×1 convolution operation to reduce the channel dimension of the input features, thereby ensuring that the overall module remains lightweight. Subsequently, in order to achieve an enlarged receptive field, a sequence of operations involving convolution with a stride of 2 and a max pooling layer are employed to successively downsize the spatial dimensions. The preceding feature map is then upsampled to restore the original spatial size, with the inclusion of a local skip connection at this stage. In the final stage, the features undergo a 1×1 convolution to recover the channel dimension, followed by the application of the Sigmoid activation function to introduce nonlinearity to the module. To minimize information loss, a dual-path skip connection strategy is adopted. The overall architecture is illustrated in Figure 2.

Numerous experiments have demonstrated the efficacy of global average pooling or global max pooling in capturing global information for advanced computer vision tasks [31,35]. However, these pooling techniques have limitations when applied to low-level computer vision tasks, such as image de-fogging and image de-noising. The primary reason is that while average pooling may enhance the peak signal-to-noise ratio (PSNR) in SISR reconstruction, the resulting reconstructed image conspicuously lacks high-frequency information, including structural details, textures, and edges.

**Figure 3:**
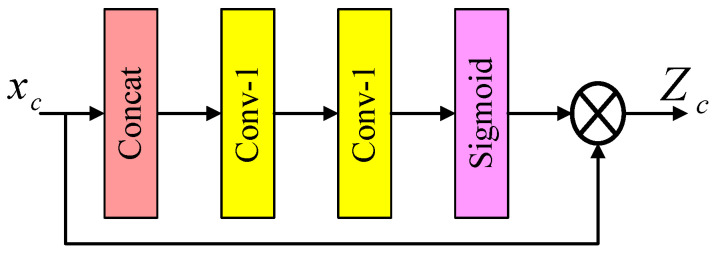
Schematic diagram of CCA.

To address this limitation, the contrast-aware channel attention (CCA) mechanism [18] deviates from traditional channel attention methods that utilize global average pooling to calculate channel weights. Instead, this attention mechanism adjusts weights based on comparative information, including the sum of the standard deviation and the mean, to determine the attention weight for each channel. This weight-assignment approach aligns more closely with the underlying logic of the visual task at hand and represents a novel contribution introduced and applied for the first time in the IMDN. The overall structure of the CCA is illustrated in Figure 3, and its mathematical formulation is presented as follows:

(2) $$Z_{c}=H_{GC}\left(x_{c}\right)=\sqrt{\frac{1}{HW}\sum_{\left(i,j\right)\in x_{c}}{\left(x_{c}^{i,j}-\frac{1}{HW}\sum_{\left(i,j\right)\in x_{c}}x_{c}^{i,j}\right)}^{2}}+\frac{1}{HW}\sum_{\left(i,j\right)\in x_{c}}x_{c}^{i,j}$$

where $x_{c}$ represents the feature map of the *c*th input, $H_{GC}\left(\right)$ represents the global contrast (GC) information evaluation function, *H* and *W* represent the height and width of the image, *i* and *j* represent the positions of the pixel points, and $Z_{c}$ represents the final output tensor.

#### 3.2.2. Wide-Activated Residual Feature Distillation

**Figure 4:**
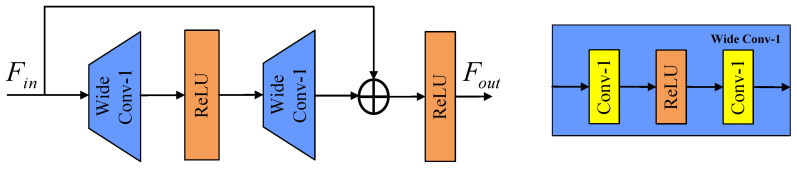
Schematic diagram of wide convolution.

The fundamental concept behind wide activation is to expand the input features before applying the activation function, aiming to enhance the model’s sensitivity to features across different magnitude ranges. By allowing more information to pass through the activation function, the model can better capture the diverse characteristics of the input. The specific implementation of wide activation is illustrated in Figure 4, typically performed by widening the dimensionality of the feature channel. In the context of SISR, wide activation has gained significant attention, due to its straightforward implementation and notable improvement in reconstruction outcomes. WDSR was initially introduced in SISR, demonstrating that the ReLU activation function yields the most favorable results. Additionally, OverNet proposes a residual module based on wide activation, which facilitates the comprehensive extraction and learning of image features by the network. Overall, wide activation in SISR involves expanding the input features prior to applying the activation function, leading to enhanced responsiveness to features across different magnitude ranges. This technique has been widely embraced in various SISR models, showcasing compelling improvements in image reconstruction outcomes.

**Figure 5:**
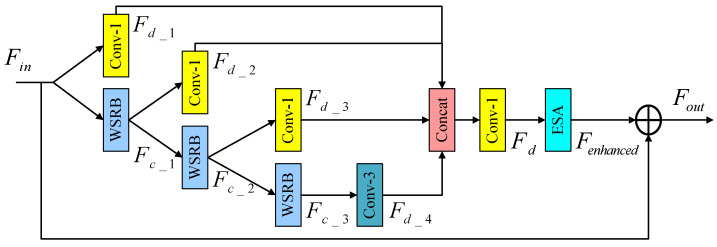
Schematic diagram of wide-activate residual feature distillation block.

In addition, taking inspiration from the residual feature distillation block proposed by Liu et al. within the lightweight residual feature distillation network model (RFDN) [21], this study advances the concept of fine-grained residual learning and introduces a feature distillation module that incorporates wide-activation and attention mechanisms. The module encompasses a distillation module for progressive refinement, an attention mechanism, and a 1×1 convolution operation employed to reduce the number of feature channels. Figure 5 presents the wide-activate residual feature distillation block (WRFDB), illustrating the complete residual structure. Notably, the module consists of three distinct stages: feature distillation, feature concentration, and feature enhancement.

The first stage is the feature distillation stage, which consists of multiple distillation layers, and the extracted features are simultaneously fed into the left and right branches for different processing. The role of the left branch is to retain the extracted features, and the role of the right branch is to further refine the features through the subsequent distillation layer. Given the input feature Fin, the specific process is described as follows:

(3) $$F_{d\_1},F_{c\_1}=DL_{1}\left(F_{in}\right),RL_{1}\left(F_{in}\right)$$

(4) $$F_{d\_2},F_{c\_2}=DL_{2}\left(F_{c\_1}\right),RL_{1}\left(F_{c\_1}\right)$$

(5) $$F_{d\_3},F_{c\_3}=DL_{3}\left(F_{c\_2}\right),RL_{1}\left(F_{c\_2}\right)$$

(6) $$F_{d\_4}=DL_{4}\left(F_{c\_3}\right)$$

where $DL_{i}\left(\right)$ represents the distillation layer, which is used to generate the distillation characteristics; $RL_{i}\left(\right)$ stands for refinement layer, which can further handle coarse-grained features; and *i* represents the *i*th layer of the distillation stage.

Secondly, in the feature concentration stage, the distillation features $F_{d\_1}$, $F_{d\_2}$, $F_{d\_3}$, and $F_{d\_4}$ are Concat, and then a 1×1 convolution layer is used for channel compression and dimension reduction:

(7) $$F_{d}=F_{1\times 1}\left(Concat\left(F_{d\_1},F_{d\_2},F_{d\_3},F_{d\_4}\right)\right)$$

where Concat() represents the addition operation along the channel dimension; $F_{d\_1}$, $F_{d\_2}$, $F_{d\_3}$, and $F_{d\_4}$ all represent the compressed features; $F_{1\times 1}$ represents the convolution layer with convolution kernel size 1; and $F_{d}$ represents feature information obtained through a series of operations.

Finally, in the feature enhancement stage, in order to improve the feature expression ability of the model, ESA is introduced, and $F_{d}$ as input is transmitted to the ESA, which enhances the representation ability of the model from the perspective of the spatial domain:

(8) $$F_{enhanced}=H_{ESA}\left(F_{d}\right)$$

where $H_{ESA}\left(\right)$ represents the ESA and $F_{enhanced}$ represents the enhanced output features.

### 3.3. Gated Feature Fusion Unit

The generation of distinct weights based on variations in feature information can enhance the utilization of effective feature information in reconstructed images [47]. Adaptive fusion of feature information at different levels holds potential for advancing various visual tasks. For instance, Lin et al. [48] employed feature pyramids to merge high-level semantic feature maps across multiple scales for object detection. Similarly, He et al. [49] leveraged dense connectivity to maximize information utilization at all levels in the context of image de-fogging.

To address the issue of information redundancy arising from indiscriminate element-wise addition operations, this study introduces a gated feature fusion module within the network model. The module takes as input the concatenation of $F_{1}$ and $F_{2}$ and produces an output with two channels. The feature information from the two branches is weighted using adaptive weights learned during training. The module can be defined as follows:

(9) $$\left(M_{1},M_{2}\right)=G\left(F_{1},F_{2}\right)$$

(10) $$F_{0}=M_{1}*F_{1}+M_{2}*F_{2}$$

where G() represents the gated fusion function; $F_{1}$ and $F_{2}$ represent the high-frequency feature information extracted by the upper and lower branches and input it into the gated feature fusion module; and $M_{1}$ and $M_{2}$ represent the weight of the corresponding channel output of the module. Finally, the feature graph $F_{1}$ and $F_{2}$ of the upper and lower branches are linearly combined with the learned weight, respectively; $F_{0}$ represents the fused feature.

### 3.4. Image Reconstruction

**Figure 6:**
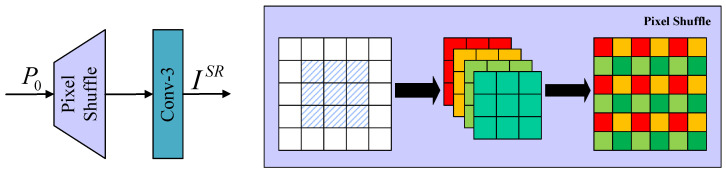
Schematic diagram of image reconstruction module. The blue shaded area means the central image block, while the white area denotes the surrounding image blocks. The colors red, orange, cyan, and grass green each represent different convolution kernels.

In the ultimate stage of image reconstruction, the upsampling reconstruction module incorporates subpixel convolution and a 3×3 convolution layer. The upsampling reconstruction module is illustrated in Figure 6; the subpixel convolution is specifically devised to upsample and expand the feature map, followed by the utilization of the final convolution layer to generate high-resolution reconstruction results with an output channel count of 3. The output can be represented as follows:

(11) $$I^{SR}=f_{up}\left(P_{0}\right)$$

where, $f_{up}$ represents the upsample reconstruction function, $P_{0}$ represents the output after the fusion of deep feature module and shallow feature, and $I^{SR}$ represents the final reconstruction result.

## 4. Experimental Results and Analysis

### 4.1. Experimental Settings

The study utilized the DIV2K dataset, which consists of high-definition natural images and their corresponding low-resolution counterparts obtained through interpolation. For training purposes, 800 images from this dataset were selected. These images exhibit clear and intricate texture details, making them suitable for training in the field of natural SISR. To assess the performance of the proposed model, benchmark test sets such as Set5 [50], Set14 [51], BSD100 [52], and Urban100 [53] were employed. These datasets served as standardized benchmarks for evaluating the effectiveness of the model. The experimental hardware environment for this training test included an Intel(R) Core(TM) i9-10980XE CPU@ 3.00 GHz with 18 cores and 36 threads. The system memory was 64 GB, and the GPU used was NVIDIA RTX 3090 with 24 GB of video memory. The software environment utilized was the Ubuntu 20.04 OS.

$L_{1}$ loss was used as a loss function to optimize the difference between the reconstructed high-resolution image and the real high-resolution image, and θ was the set of parameters in the network that needed to be learned. The loss function can be expressed as follows:

(12) $$L\left(\theta \right)=\frac{1}{N}\sum_{i=1}^{N}{∥I_{HR}^{i}-I_{SR}^{i}∥}_{1}$$

### 4.2. Comparisons with Other Algorithms

#### 4.2.1. PSNR and SSIM Comparisons

To further verify the validity of our WFDN, it was compared with mainstream classical models, such as Bicubic [9], the ESPCN (CVPR 2016) [15], VDSR (CVPR 2016) [13], the DRCN (CVPR 2016) [24], the LapSRN (TPAMI 2017) [14], WDSR-Mini (CVPR 2018) [22], the IMDN (ACM MM 2019) [18], SMSR (CVPR 2021) [27], ESRT (CVPR 2022) [38], the ARRFN (NC 2022) [54], LBNet (IJCAI 2022) [55], LatticeNet (TPAMI 2022) [56], the MFRN (IEEE Access 2023) [57], NGSwin (CVPR 2023) [58], VapSR (ECCV 2023) [59], and the PILN (TII 2023) [60]. The super-resolution reconstruction results of the Set5 [50], Set14 [51], BSD100 [52], and Urban100 [53] test sets under different magnification factors (×2, ×3, ×4) were quantitatively compared and qualitatively analyzed, respectively, as shown in Table 1.

As can be seen from Table 1, the reconstruction metrics of our WFDN are significantly better than the other models. In addition, each branch of the WFDN model proposed in this paper is constructed by the combination of four wide-activated residual feature distillation modules. The reconstruction results of different magnifications were tested and compared on each test set. The experimental results show that the proposed model is superior to the WDSR-Mini network [22] model composed of 16 wide-active residuals in the PSNR and the SSIM. When the magnification was 2, compared with the LatticeNet algorithm, the network model only had a slight gap in the objective index PSNR of a few datasets, but it had the highest objective evaluation index in the other test sets. In particular, the average PSNR on the BSD100 and Urban100 data sets was 0.08 dB and 0.36 dB higher than that of the LatticeNet network model, and the SSIM value was increased by 0.005 and 0.010, respectively. When the magnification was 4, compared with Bicubic, the ESPCN, VDSR, the DRCN, the LapSRN, WDSR-Mini, the IMDN, and LatticeNet, the PSNR value of the proposed algorithm in the Set5 test set increased by 4.06 dB, 1.82 dB, 1.13 dB, 0.95 dB, 0.94 dB and 0.31 dB, 0.27 dB, and 0.18 dB, respectively. The WFDN had the best reconstruction performance when the magnification was 4, which was higher than the other algorithms. In summary, our WFDN had good reconstruction performance under different magnifications.

#### 4.2.2. Visual Performance Comparisons

**Figure 7:**
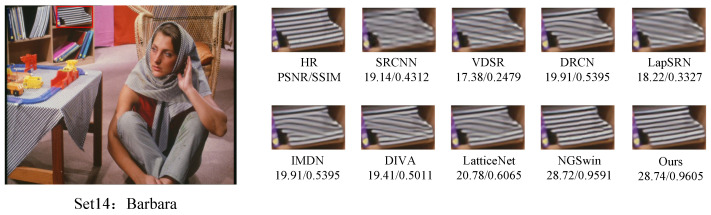
Comparisons of reconstruction effects by different methods on the barbara.png image in Set5, where the red box represents the selected block of pixels.

**Figure 8:**
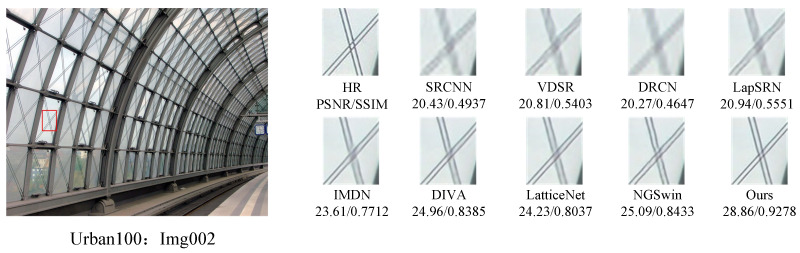
Comparisons of reconstruction effects by different methods on the img002.png image in Urban100, where the red box represents the selected block of pixels.

**Figure 9:**
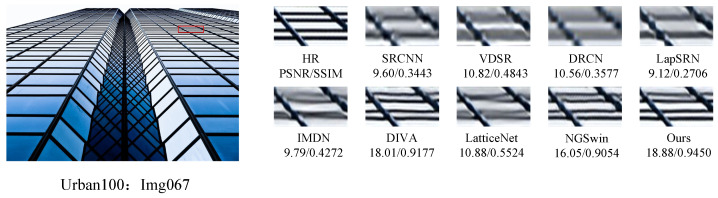
Comparisons of reconstruction effects by different methods on the img067.png image in Urban100, where the red box represents the selected block of pixels.

**Figure 10:**
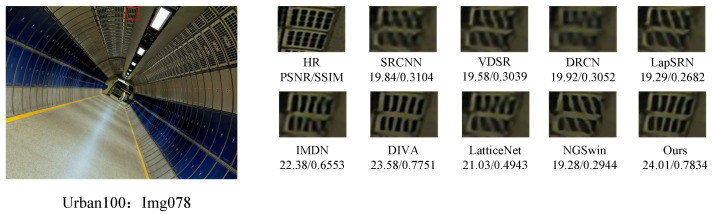
Comparisons of reconstruction effects by different methods on the img078.png image in Urban100, where the red box represents the selected block of pixels.

In order to further demonstrate the relative superiority of the reconstruction results of the proposed algorithm, the visual effects of different reconstruction methods at a magnification of 4 were emphasized, and the local area of the image was amplified, as shown in Figure 7, Figure 8, Figure 9 and Figure 10. The PSNR and SSIM values of the reconstructed images by each algorithm are shown in the figure below.

Figure 7 presents the results of reconstructing the barbara.png image from the Set5 dataset, using various methods. It can be observed that the reconstructions from the SRCNN, VDSR, and LapSRN models exhibit relatively blurry effects. In comparison to the original image, the reconstruction from the DRCN model lacks pixels, resulting in unclear textures. The reconstructions from the IMDN and LatticeNet models show overly distorted lines. In contrast, the reconstructions from our proposed model better preserve the overall shape and clarity of the stripes in the image. Figure 8 displays the results of reconstructing the img002 image from the Urban100 dataset using different methods. It can be seen from the reconstructions produced by various algorithms that the SRCNN, VDSR, DRCN, and LapSRN models performed poorly in handling details, resulting in blurry textures. Our proposed method and NGSwin, however, exhibit superior subjective visual performance, with clearer and more distinct details. Figure 9 shows the reconstructions of the building image numbered 67 from the Urban100 dataset. The reconstructions from the SRCNN, VDSR, the DRCN, and the LapSRN exhibit unclear overall contours of window frames, blurry backgrounds, and lack of texture. In contrast, the reconstructions from DIVA and NGSwin show significant improvements, with clearer details in the window frames and no distortion in the lines. However, the overall image is relatively dark and exhibits slight distortion. In the reconstructions from our algorithm, the contours of the window lines are clear, and the overall clarity of the image is enhanced, to a certain extent. Figure 10, taken from the Urban100 dataset, demonstrates that our proposed algorithm significantly alleviates the problem of overall darkness in the reconstructions produced by the SRCNN, the DRCN, and the LapSRN. Lines reconstructed by LatticeNet and NGSwin exhibit distortion. Compared to the reconstructions from the IMDN and DIVA algorithms, the window lines in the buildings show varying degrees of curvature. Our model effectively preserves the structural texture of the buildings, with straighter edges in the window lines. Therefore, our WFDN shows improvements in objective evaluation metrics, yielding reconstructed images with richer texture details, more realistic lines, and clearer structures in subjective visual assessment.

#### 4.2.3. MOS Comparisons

The mean opinion score (MOS) is a widely adopted subjective evaluation metric in the field of visual tasks, extensively employed on an international scale. This metric entails selecting a representative group of individuals, including both experts and non-experts, in appropriate proportions, and soliciting their assessment of provided images. The evaluation criterion primarily hinges upon the visual comfort experienced by human observers during the image observation process. Following the exclusion of extreme scores, the remaining scores are averaged in a descending order to derive the final MOS result.

As illustrated in Table 2, our algorithm exhibits superior performance in the majority of cases, achieving the top-ranking position in the MOS test. Additionally, it achieved a second-place performance in certain scenarios. This outcome not only provides substantial evidence of our WFDN’s ability to generate visually pleasing and natural images, but also highlights its robustness and proficiency in effectively recovering the diverse range of textures present in the datasets.

#### 4.2.4. Comparisons with the Benchmark Model IMDN

**Figure 11:**
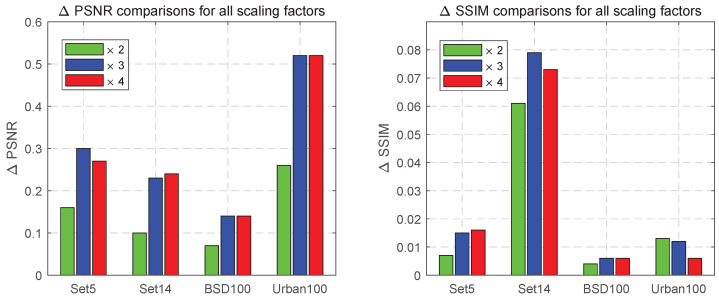
Comparisons with the benchmark model IMDN ×2, ×3, and ×4 tests in Set5, Set14, BSD100, and Urban100. The objective evaluation metrics were ΔPSNR or ΔSSIM. The test results are presented as histograms with the names of the datasets directly below them.

The IMDN is widely recognized as the benchmark algorithm for SISR, and its effectiveness was evaluated using quantified metrics such as the PSNR and the SSIM. The histogram presented in Figure 11 depicts the ΔPSNR or ΔSSIM values between our WFDN and the IMDN benchmark. The results consistently demonstrate that the WFDN outperformed the IMDN across all four widely recognized texture datasets, with exceptional performance on more challenging ones like BSD100 and Urban100. These findings firmly establish the WFDN’s superior texture restoration capabilities compared to the IMDN.

#### 4.2.5. Comparisons with the Recurrent-Based Methods

**Figure 12:**
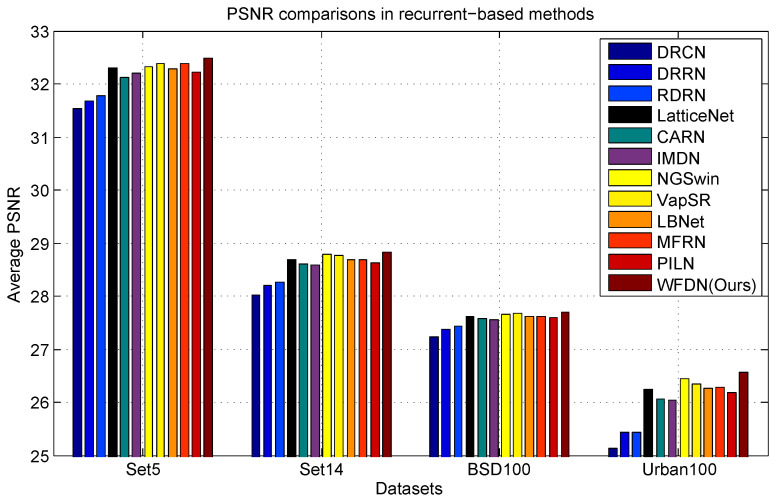
×4 SISR performance comparisons of recurrent-based algorithms. The objective evaluation metric was the PSNR, and Set5, Set14, BSD100, and Urban100 were the test datasets.

Given that our WFDN is based on a recurrent network, it is crucial to provide a concise overview of recurrent algorithms. Recurrent algorithms are specifically designed to reduce training time by enabling module re-use and effectively mitigating the loss of features during information transmission. This ensures optimal information utilization throughout the network. In this subsection, we present a comprehensive comparison of recurrent networks from one perspective: namely, network performance. To facilitate fair comparisons, we evaluated our WFDN against 11 prominent SISR recurrent networks: namely, the DRCN, the DRRN, the RDRN, LatticeNet, the CARN, the IMDN, NGSwin, VapSR, LBNet, the MFRN, and the PILN. The evaluation results are depicted in Figure 12. The observations reveal that our WFDN achieved the highest reconstruction performance among all the considered recurrent networks.

### 4.3. Ablation Experiments on Network Modules

The core foundation of super-resolution models lies in shallow feature extraction, high-frequency feature extraction, and image reconstruction, and the importance of these key steps has been widely verified. Their absence will seriously impair the performance of the model and even lead to catastrophic consequences. Therefore, the ablation experiments in this chapter focused on the consideration of the number of wide-activation modules and the gated fusion unit, in order to deeply explore the impact of these elements on the model performance. For the ablation experiments, we chose Urban100 [36] as the test set, because the image textures in [36] are very complex and not easy to deal with, so the results of different algorithms are significantly different. Meanwhile, the scale factor was 4.

As evidenced in Table 3 and Table 4, the wide-activation unit and the gate unit are both integral components of the model. The wide-activation unit exhibited a favorable trade-off with regard to space complexity, efficiently facilitating the calibration and updating of the parameters, ultimately leading to enhanced model performance. Furthermore, the gate unit seamlessly integrated linear and nonlinear features, enriching the feature representation and augmenting the model’s capacity to learn discriminative features, thereby elevating its overall performance.

### 4.4. Ablation Experiments on Distillation Blocks with Wide-Activation Residual Features

**Figure 13:**
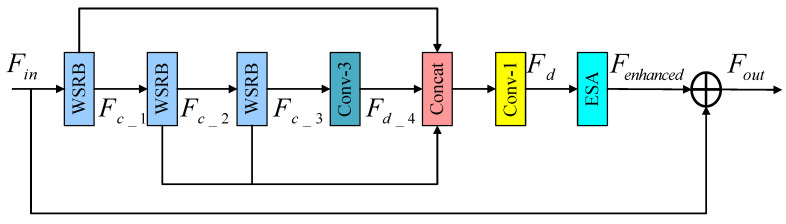
Schematic structure of WRFDB.

The WRFDB (wide-activate residual feature distillation block) served as the fundamental unit for network feature extraction in this study. This section primarily focuses on validating the efficacy of the channel-compression step within the residual feature distillation block. In contrast to Figure 5, the left portion of the distillation layer in Figure 13 omitted the utilization of a 1×1 convolution layer for channel compression. Instead, the convolution layer was removed, and the features extracted by the WSRB were directly concatenated. The test results are presented in Table 1.

Observing the outcomes in Table 5, it is evident that both variations of the WRFDBs exhibited comparable super-resolution reconstruction performance, except for a slight difference in the PSNR value on the Set5 dataset. Notably, the model with the channel-compression function achieved higher PSNR values, surpassing the corresponding model on the Set14, BSD100, and Urban100 datasets by 0.06dB, 0.02dB, and 0.08dB, respectively. While reducing the channel-compression steps may result in a more concise model, the experimental findings demonstrate a significant decline in the overall reconstruction effectiveness. Hence, the inclusion of channel-compression steps effectively enhances the network’s feature extraction capability.

**Figure 14:**
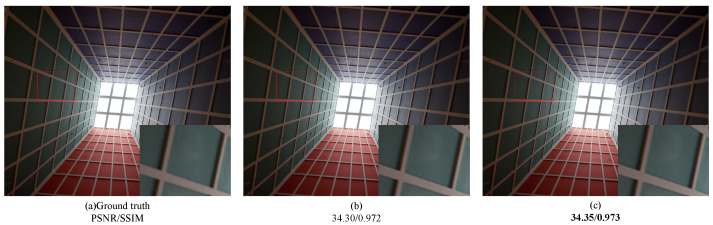
Ablation experiments on wide-activation residual feature distillation modules lacking channel compression, where the portion selected by the red border is the area of pixels comparing the PSNR and SSIM metrics.

To further substantiate the necessity of the channel-compression step in the feature distillation block, we selected the Img090 images from the Urban100 dataset to visualize the reconstructed images, using two models, as depicted in Figure 14. In the figure, (a) represents the original high-definition image, (b) represents the network reconstruction image without the channel-compression step, and (c) represents the network reconstruction image with channel compression. In the bottom-right corner of the figure, we provide localized enlarged images of the original and reconstructed images, along with the corresponding PSNR and SSIM values displayed below. Upon subjective visual inspection, it becomes evident that the reconstructed texture brightness in Figure 14c appears deeper compared to Figure 14b Moreover, the intersection of lines in Figure 14c exhibits higher fidelity. Thus, the overall reconstruction effect of the image is superior when the channel-compression step is included.

### 4.5. Ablation Experiments on Attention Mechanisms

This section delves into a detailed analysis of the impacts of ESA, CCA, and their combinations on the performance of super-resolution reconstruction. A comparison is also made against existing classical attention mechanisms. The experimental results are presented in Table 6. In all four test sets, the network model integrated with attention mechanisms consistently outperformed the network model without integrated attention mechanisms, as evidenced by the higher PSNR values. This substantiates the effectiveness of the proposed attention mechanisms in enhancing the reconstruction performance of the network. Specifically, the network model in this study, which incorporated the ESA, surpassed the SENet [32] attention mechanism employed in the Set5, Set14, BSD100, and Urban100 datasets. A closer examination of the table reveals that the network’s utilization of the increased spatial attention mechanism slightly trailed behind the ESA and CCA combination on the Set5 and Set14 datasets, which contained fewer images. However, on the more challenging BSD100 and Urban100 datasets, it improved by 0.01 dB and 0.05 dB, respectively. Furthermore, the ESA alone demonstrated notably superior performance compared to the CCA. This strongly supports the superiority of integrating the ESA into the network.

### 4.6. Ablation Experiments with Activation Functions

The primary role of an activation function is to enhance the network’s nonlinear characteristics, thereby greatly improving its feature characterization ability. In this study, the WRFDB, depicted in Figure 13, served as the pivotal basic module during the feature extraction stage of the network model. This section conducts a thorough comparative analysis of three commonly used activation functions in the field of super-resolution reconstruction. As observed in Table 7, the objective indicators of the LeakyReLU [61] activation function in the BSD100 and Urban100 test sets were not optimal; nonetheless, the results in both test sets were only slightly lower than the optimal values by 0.01 dB and 0.04 dB, respectively. Notably, the LeakyReLU activation function demonstrated the best performance, in terms of the objective indicator PSNR in the Set5 and Set14 test sets. Comparing it to the ReLU [62] and GELU [63] activation functions, the LeakyReLU activation function exhibited significant improvements in the PSNR, with increases of 0.03 dB, 0.08 dB, 0.04 dB, and 0.04 dB, respectively. From these findings, it can be concluded that the LeakyReLU activation function performs adequately on large datasets while significantly enhancing the reconstruction effect on smaller datasets. Consequently, after comprehensive consideration, the network ultimately adopts the LeakyReLU activation function for super-resolution reconstruction, aiming to achieve superior results.

### 4.7. Ablation Experiments on the Speed of the Model

The speed of a model is one of the key measures of its performance. This paper focused on evaluating the average running time of different models when processing the Urban100 dataset containing 100 images, especially in the case of ×4 SISR results. Although the resolution of the images in the urban dataset varies, they are all high-definition images. The results we derived were based on the average computing speed of each model for processing these 100 images. See Table 8.

It can be seen that the distillation model achieved efficient operation speed while maintaining details. Although our algorithm was slightly slower than the IMDN model, its performance was significantly improved, mainly due to the clever use of wide-activation feature extraction and fusion architecture.

## 5. Conclusions

This paper introduces a novel dual-path SISR reconstruction model based on the wide-activation feature distillation network (WFDN). The model leverages a residual network as the backbone to construct a dual-path parallel structure, enhancing feature utilization through global residual connectivity. In the deep feature mapping stage, the residual feature distillation module is further improved by integrating the attention mechanism and the wide activation concept. This integration enables the attention mechanism to suppress redundant information and focus on high-frequency spatial details. Additionally, the wide-activation residual block enriches feature extraction diversity by expanding the network width and reducing the number of parameters and the memory consumption. In the subsequent feature fusion stage, a gated fusion mechanism is employed to mitigate information redundancy resulting from the addition of undifferentiated elements. This mechanism dynamically assigns different weights to the deep feature information extracted from the two branches, enabling effective capture of the final high-frequency features through weighted fusion. The proposed network model yields impressive results, in terms of objective metrics, and it significantly enhances the details of the reconstructed images, demonstrating superiority over popular existing models.

﻿

## Figures and Tables

**Table 1:** Reconstruction results comparison of the baseline datasets at magnifications of 2, 3, and 4. The best and the second-best results are in **bold** and underline, respectively.

Model	Scale	Para	FLOP	Set 5 [50]	Set 14 [51]	BSD 100 [52]	Urban 100 [53]
				PSNR/SSIM	PSNR/SSIM	PSNR/SSIM	PSNR/SSIM
Bicubic [9]	×2	-	-	36.66/0.929	30.24/0.868	29.56/0.843	26.88/0.840
ESPCN (CVPR 2016) [15]		-	-	37.00/0.956	32.75/0.910	31.51/0.894	29.87/0.907
VDSR (CVPR 2016) [13]		0.66 M	612.6 G	37.54/0.959	33.03/0.912	31.90/0.896	30.76/0.914
DRCN (CVPR 2016) [24]		1.77 M	17,974.0 G	37.63/0.959	33.06/0.912	31.85/0.884	30.76/0.913
LapSRN (TPAMI 2017) [14]		0.81 M	29.9 G	37.52/0.959	32.99/0.912	31.80/0.895	30.45/0.913
CARN(ECCV 2018) [26]		1.59 M	222.8 G	37.76/0.959	33.52/0.916	32.09/0.897	31.92/0.925
WDSR-Mini (CVPR 2018) [22]		-	-	37.96/0.960	33.50/0.914	32.13/0.899	31.79/0.921
IMDN (ACM MM 2019) [18]		0.69 M	158.8 G	38.00/0.961	33.63/0.912	32.19/0.900	32.17/0.928
SMSR (CVPR 2021) [27]		0.98 M	131.6 G	38.00/0.960	33.64/0.918	32.17/0.900	32.19/0.928
ESRT(CVPR 2022) [38]		-	-	38.07/0.961	33.62/0.919	**32.29**/0.901	**32.59**/0.932
ARRFN (NC 2022) [54]		0.98 M	-	38.01/0.960	33.66/0.917	32.20/0.899	32.27/0.929
LBNet (IJCAI 2022) [55]		-	-	38.05/0.961	33.65/0.918	32.15/0.900	32.30/0.928
LatticeNet (TPAMI 2022) [56]		0.75 M	169.0 G	38.15/0.961	33.78/0.919	32.25/0.901	32.43/0.930
MFRN (IEEE Access 2023) [57]		-	-	38.04/0.961	33.67/0.918	32.21/0.900	32.28/0.930
NGSwin (CVPR 2023) [58]		1.00 M	140.4 G	38.05/0.961	**33.79**/0.919	32.27/0.900	32.53/0.932
VapSR (ECCV 2023) [59]		0.33 M	-	38.08/0.961	33.77/0.919	32.27/0.901	32.45/0.931
PILN(TII 2023) [60]		0.58 M	-	38.08/0.960	33.72/0.918	32.23/0.900	32.38/0.930
WFDN (Ours)		0.78 M	163.7 G	**38.16**/**0.968**	33.73/**0.973**	32.26/**0.904**	32.43/**0.941**
Bicubic [9]	×3	-	-	30.39/0.868	27.55/0.774	27.21/0.738	24.46/0.735
ESPCN (CVPR 2016) [15]		-	-	33.02/0.914	29.49/0.827	28.50/0.794	26.41/0.816
VDSR (CVPR 2016) [13]		0.66 M	613.0 G	33.65/0.921	29.78/0.831	28.82/0.798	27.14/0.828
DRCN (CVPR 2016) [24]		1.77 M	17,974.0 G	33.82/0.923	29.77/0.831	28.80/0.796	27.15/0.823
LapSRN (TPAMI 2017) [14]		-	-	33.82/0.923	29.87/0.832	28.83/0.798	27.08/0.828
CARN(ECCV 2018) [26]		1.59 M	118.8 G	34.29/0.925	30.29/0.840	29.06/0.803	28.06/0.849
WDSR-Mini (CVPR 2018) [22]		-	-	34.38/0.925	30.31/0.840	29.06/0.803	27.97/0.847
IMDN (ACM MM 2019) [18]		0.70 M	71.5 G	34.36/0.927	30.32/0.842	29.09/0.805	28.17/0.852
SMSR (CVPR 2021) [27]		0.99 M	67.8 G	34.40/0.927	30.33/0.841	29.10/0.805	28.25/0.853
ESRT(CVPR 2022) [38]		0.77 M	-	34.42/0.926	30.43/0.843	29.15/0.806	28.46/0.857
ARRFN (NC 2022) [54]		0.99 M	-	34.38/0.927	30.36/0.842	29.09/0.805	28.22/0.853
LBNet (IJCAI 2022) [55]		0.73 M	-	34.47/0.927	30.38/0.841	29.13/0.806	28.42/0.855
LatticeNet (TPAMI 2022) [56]		0.76 M	76.0 G	34.53/0.928	30.39/0.842	29.15/0.806	28.33/0.854
MFRN (IEEE Access 2023) [57]		-	-	34.49/0.928	30.39/0.842	29.14/0.807	28.45/0.856
NGSwin (CVPR 2023) [58]		1.01 M	66.6 G	34.52/0.928	30.53/0.845	29.19/0.807	28.52/0.860
VapSR (ECCV 2023) [59]		0.33 M	-	34.52/0.928	30.53/0.845	29.19/0.807	28.43/0.858
PILN(TII 2023) [60]		0.59 M	-	34.39/0.926	30.34/0.841	29.08/0.804	28.09/0.850
WFDN (Ours)		0.80 M	72.6 G	**34.66**/**0.942**	**30.55**/**0.921**	**29.23**/**0.811**	**28.69**/**0.864**
Bicubic [9]	×4	-	-	28.42/0.810	26.00/0.703	25.96/0.668	23.14/0.657
ESPCN (CVPR 2016) [15]		-	-	30.66/0.865	27.71/0.756	26.98/0.712	24.60/0.736
VDSR (CVPR 2016) [13]		0.66 M	613.0 G	31.35/0.884	28.01/0.767	27.30/0.725	25.18/0.751
DRCN (CVPR 2016) [24]		1.77 M	17,974.0 G	31.53/0.885	28.03/0.767	27.24/0.723	25.14/0.751
LapSRN (TPAMI 2017) [14]		0.81 M	149.4 G	31.54/0.886	28.19/0.772	27.33/0.726	25.21/0.756
CARN(ECCV 2018) [26]		1.59 M	90.9 G	32.13/0.893	28.60/0.780	27.58/0.734	26.07/0.783
WDSR-Mini (CVPR 2018) [22]		-	-	32.17/0.893	28.59/0.783	27.56/0.731	25.95/0.778
IMDN (ACM MM 2019) [18]		0.70 M	40.9 G	32.21/0.895	28.58/0.787	27.56/0.735	26.04/0.784
SMSR (CVPR 2021) [27]		1.00 M	41.6 G	32.12/0.893	28.55/0.780	27.55/0.735	26.11/0.786
ESRT(CVPR 2022) [38]		0.75 M	-	32.19/0.894	28.69/0.783	27.69/0.737	26.39/**0.796**
ARRFN (NC 2022) [54]		1.00 M	-	32.22/0.895	28.60/0.781	27.57/0.735	26.09/0.785
LBNet (IJCAI 2022) [55]		0.74 M	-	32.29/0.896	28.68/0.783	27.62/0.738	26.27/0.790
LatticeNet (TPAMI 2022) [56]		0.78 M	43.0 G	32.30/0.896	28.68/0.783	27.62/0.737	26.25/0.787
MFRN (IEEE Access 2023) [57]		-	-	32.39/0.896	28.69/0.784	27.62/0.739	26.29/0.791
NGSwin (CVPR 2023) [58]		1.02 M	36.4 G	32.33/0.896	28.78/0.785	27.66/0.739	26.45/**0.796**
VapSR (ECCV 2023) [59]		0.34 M	-	32.38/0.897	28.77/0.785	27.68/0.739	26.35/0.794
PILN(TII 2023) [60]		0.60 M	-	32.22/0.894	28.62/0.781	27.59/0.736	26.19/0.787
WFDN (Ours)		0.82 M	48.2 G	**32.48**/**0.911**	**28.82**/**0.860**	**27.70**/**0.741**	**26.56**/0.790

**Table 2:** MOS comparisons. Top 5 algorithms for scale factor ×2, ×3, ×4 on datasets Set5, Set14, BSD100, and Urban100. Our WFDN is shown in **bold**.

Dataset	Scale	Top 5 Algorithms
Set5 [50]	×2	**WFDN** > VapSR > NGSwin > MFRN > PILN
Set5 [50]	×3	**WFDN** > VapSR > MFRN > NGSwin > LBNet
Set5 [50]	×4	**WFDN** > MFRN > VapSR > NGSwin > LBNet
Set14 [51]	×2	VapSR > **WFDN** > MFRN > PILN > NGSwin
Set14 [51]	×3	**WFDN** > MFRN > VapSR > NGSwin > PILN
Set14 [51]	×4	**WFDN** > MFRN > VapSR > NGSwin > PILN
BSD100 [52]	×2	**WFDN** > VapSR > MFRN > PILN > LBNet
BSD100 [52]	×3	**WFDN** > VapSR > MFRN > NGSwin > PILN
BSD100 [52]	×4	**WFDN** > MFRN > NGSwin > VapSR > LBNet
Urban100 [53]	×2	**WFDN** > VapSR > NGSwin > MFRN > PILN
Urban100 [53]	×3	**WFDN** > MFRN > NGSwin > VapSR > LBNet
Urban100 [53]	×4	**WFDN** > MFRN > NGSwin > VapSR > LBNet

**Table 3:** Ablation experiments with a wide-activation block number, where FLOPs represents the number of floating point operations, Para is the size of the parameters, and Running time means the running time of the model. The best results are in **bold**.

Block Number	FLOPs (G)	Running Time (ms)	Para (M)	PSNR (dB)/SSIM
1	40.9	**38**	**0.72**	26.04/0.784
2	44.6	40	0.73	26.13/0.785
3	40.1	45	0.76	26.24/0.786
4	**48.2**	47	0.82	**26.56**/**0.790**

**Table 4:** Ablation experiments with gated fusion, where FLOPs represents the number of floating point operations, Para is the size of the parameters, and Running time means the running time of the model. The best results are in **bold**.

Gate Fusion	FLOPs (G)	Running Time (ms)	Para (M)	PSNR (dB)/SSIM
×	47.3	**45**	**0.80**	26.33/0.785
✓	**48.2**	47	0.82	**26.56**/**0.790**

**Table 5:** Ablation experiments with WRFDB modules on ×2 SISR performance; the best results are in **bold**.

WRFDB	Set5 [50] PSNR/dB	Set14 [51] PSNR/dB	BSD100 [52] PSNR/dB	Urban100 [53] PSNR/dB
×	**38.13**	33.66	32.25	32.40
✓	**38.13**	**33.72**	**32.27**	**32.48**

**Table 6:** Effects of attention mechanisms on ×2 SISR performance. The best results are in **bold**.

Attention Mechanisms	Set5 [50] PSNR/dB	Set14 [51] PSNR/dB	BSD100 [52] PSNR/dB	Urban100 [53] PSNR/dB
×	38.10	33.68	32.24	32.28
SE [32]	38.13	33.68	32.23	32.39
ESA [36]	38.13	33.72	**32.27**	**32.48**
CCA [18]	38.13	33.70	32.21	32.31
ESA + CCA	**38.16**	**33.73**	32.26	32.43

**Table 7:** Effects of activation function on ×2 SISR performance. The best results are in **bold**.

Activation Functions	Set5 [50] PSNR/dB	Set14 [51] PSNR/dB	BSD100 [52] PSNR/dB	Urban100 [53] PSNR/dB
ReLU [62]	**38.13**	33.65	**32.27**	32.47
GELU [63]	38.12	33.69	32.26	32.40
LeakyReLU [61]	**38.13**	**33.72**	**32.27**	**32.48**

**Table 8:** Ablation experiments on the model’s running time (ms). The best results are in **bold**.

Method	Time	Method	Time	Method	Time	Method	Time	Method	Time
SRCNN	297	LapSRN	189	EDSR	315	CARN	335	IMDN	**38**
SMSR	309	ARRFN	234	LBNet	679	MFRN	361	WFDN	47

## Data Availability

The data presented in this study are available on request from the corresponding author.
